# Development of models for classification of action between heat-clearing herbs and blood-activating stasis-resolving herbs based on theory of traditional Chinese medicine

**DOI:** 10.1186/s13020-018-0169-x

**Published:** 2018-02-27

**Authors:** Zhao Chen, Yanfeng Cao, Shuaibing He, Yanjiang Qiao

**Affiliations:** 1 School of Chines Materia Medica, Beijing University of Chinese Medicine, Yangguang South Avenue, Fangshan District, Beijing, 102488 China; 2 Research Center of TCM Information Engineering, Beijing University of Chinese Medicine, Yangguang South Avenue, Fangshan District, Beijing, 102488 China

**Keywords:** Heat-clearing herbs (HCHs), Blood-activating stasis-resolving herbs (BASRHs), Machine learning, Deep learning, Herbal property, Traditional Chinese medicine (TCM)

## Abstract

**Background:**

Action (“*gongxiao*” in Chinese) of traditional Chinese medicine (TCM) is the high recapitulation for therapeutic and health-preserving effects under the guidance of TCM theory. TCM-defined herbal properties (“*yaoxing*” in Chinese) had been used in this research. TCM herbal property (TCM-HP) is the high generalization and summary for actions, both of which come from long-term effective clinical practice in two thousands of years in China. However, the specific relationship between TCM-HP and action of TCM is complex and unclear from a scientific perspective. The research about this is conducive to expound the connotation of TCM-HP theory and is of important significance for the development of the TCM-HP theory.

**Methods:**

One hundred and thirty-three herbs including 88 heat-clearing herbs (HCHs) and 45 blood-activating stasis-resolving herbs (BAHRHs) were collected from reputable TCM literatures, and their corresponding TCM-HPs/actions information were collected from Chinese pharmacopoeia (2015 edition). The Kennard–Stone (K–S) algorithm was used to split 133 herbs into 100 calibration samples and 33 validation samples. Then, machine learning methods including supported vector machine (SVM), k-nearest neighbor (kNN) and deep learning methods including deep belief network (DBN), convolutional neutral network (CNN) were adopted to develop action classification models based on TCM-HP theory, respectively. In order to ensure robustness, these four classification methods were evaluated by using the method of tenfold cross validation and 20 external validation samples for prediction.

**Results:**

As results, 72.7–100% of 33 validation samples including 17 HCHs and 16 BASRHs were correctly predicted by these four types of methods. Both of the DBN and CNN methods gave out the best results and their sensitivity, specificity, precision, accuracy were all 100.00%. Especially, the predicted results of external validation set showed that the performance of deep learning methods (DBN, CNN) were better than traditional machine learning methods (kNN, SVM) in terms of their sensitivity, specificity, precision, accuracy. Moreover, the distribution patterns of TCM-HPs of HCHs and BASRHs were also analyzed to detect the featured TCM-HPs of these two types of herbs. The result showed that the featured TCM-HPs of HCHs were cold, bitter, liver and stomach meridians entered, while those of BASRHs were warm, bitter and pungent, liver meridian entered.

**Conclusions:**

The performance on validation set and external validation set of deep learning methods (DBN, CNN) were better than machine learning models (kNN, SVM) in sensitivity, specificity, precision, accuracy when predicting the actions of heat-clearing and blood-activating stasis-resolving based on TCM-HP theory. The deep learning classification methods owned better generalization ability and accuracy when predicting the actions of heat-clearing and blood-activating stasis-resolving based on TCM-HP theory. Besides, the methods of deep learning would help us to improve our understanding about the relationship between herbal property and action, as well as to enrich and develop the theory of TCM-HP scientifically.

**Electronic supplementary material:**

The online version of this article (10.1186/s13020-018-0169-x) contains supplementary material, which is available to authorized users.

## Background

Traditional Chinese medicines (TCM) is one of the great herbal medicine systems worldwide, which plays an important role in current health care system in many countries. In the view of TCM theory, Yin-yang and five-elements theory is the central theory, which is used to explain how the world and body work [1]. The action of TCM is the high recapitulation for its therapeutic and health-preserving effect under the guidance of TCM theory [2]. TCM-HP, is the basic property of TCM and the high recapitulation of its functional characteristics [2]. The classic concept of TCM-HPs defines four fundamental characters (cold, cool, warm and hot), five fundamental tastes (salty, sour, bitter, sweet and pungent), four toxic states (toxic, nontoxic, very toxic, and slightly toxic), 12 meridians (bladder, spleen, large intestine, stomach, small intestine, liver, lung, heart, kidney, gallbladder, xin bao or pericardium and san jiao) [3]. TCM-HP also provides strong evidence to guide the clinical application of TCM. Many bioinformatics and pharmacological approaches were applied to study TCM-HP [1, 4, 5]. The research on the relationship between TCM-HP and action has been our great concern in the field of TCM.

The strategy of studying the relationship between TCM-HP and action have changed over the past decades. TCM-HP can not only be limited to a single property, but also need to be considered as a whole [6]. An Apriori algorithm was employed for producing association rules that described the intrinsic relationships between herbal property (qi, flavor and their combinations) and herbal efficacy [7]. However, the confirmed 120 resulting rules were dispersed and single property research could hardly characterize the whole effects of TCM. Hence the four fundamental characters, five fundamental tastes, and meridians need to be as a whole. Only in this way could we reveal the relationship between action and herbal property [8]. Multidimensional property of TCM is inherent basis of multiple action and is a collection of many herbal properties that determine the characteristics for efficiency of TCM [6]. Subsequently, property combination patterns for TCM [9–11] were proposed to reveal the relationship of TCM-HP and action from a holistic view of TCM.

Machine learning and deep learning methods have been widely applied in pharmaceutical research [12–17]. In life sciences, machine learning is often used to explain phenomena that are not completely theoretically understood [13]. Deep learning allows computational models that are composed of multiple processing layers to learn representations of data with multiple levels of abstraction [18].

Research on TCM-HP and action is helpful to discover the inherent relation between TCM-HP and action of TCM [7], which can illustrate the connotation holistic view of TCM-HP theory.

TCM-HP and action of TCM are both summary of clinical practices, so the classification of actions based on the holistic concept of TCM-HP is consistent with clinical practices. Machine learning methods have been applied to TCM researches with some success, for instance, for the TCM syndrome classification [19, 20], the relationship of TCM-HP and action [1, 3, 12, 13, 21–26].

In this work, 88 HCHs and 45 BASRHs were collected as our research objects and four different artificial intelligence methods were used to develop the classification models of TCM actions based on TCM-HP theory. In order to ensure robustness, these four constructed classification models (kNN, SVM, CNN, DBN) were tested with external validation set, including 15 HCHs and 5 BASRHs.

Heat clearing herbs, which tend to have cold characters, have been found to produce some combination of antimicrobial [27], anti-toxic [28], anti-inflammatory [29, 30], antipyretic [28], antioxidant [27, 31], platelet aggregation inhibition, sedative, immunomodulatory [32], and hepatoprotective activities [33]. The HCHs own anti-inflammatory and antimicrobial effects, and their potential mechanisms of action contributing to their anti-inflammatory and antimicrobial activity may be related to their action of removing heat and counteracting toxicity [34]. *Radix Salviae Miltiorrhizae* (*Dan Shen*) is an example of BASRHs, and the compound Tanshinone IIA isolated from it could be a promising agent to improve blood viscosity and microcirculation and to prevent cardiovascular diseases [35]. Salvianolic acid B is clinically effective because of its ability to change the gene expression profile of endothelial cells thereby preventing vascular events [36]. The Blood-Activating and Stasis-Resolving herb—Chuanxiong Hort may treat headache and has potential to be an agent for treating headache [37]. Because of the important clinical significance of these two kinds of typical TCM, they can lay the foundation for elucidating the relationship between TCM-HP and action.

## Methods

### Selection of HCHs and BASRHs

A total of 133 well established HCHs and BASRHs, given in Additional file 4: Table S1, were collected from reputable TCM literatures [38, 39], which were composed of 88 HCHs and 45 BASRHs. Their TCM-HPs and actions were collected from Chinese pharmacopoeia (2015 edition). External validation set (Additional file 4: Table S1) including 15 HCHs and 5 BASRHs were collected from a reputable TCM literature [39].

### Digital representation of TCM and feature selecting for TCM-HPs

The classic concept of TCM herbal properties (TCM-HPs) defines four fundamental characters (cold, cool, neutral, warm and hot), five fundamental tastes (salty, sour, bitter, sweet and pungent), four toxic states (toxic, nontoxic, very toxic, and slightly toxic), and 12 meridians (bladder, spleen, large intestine, stomach, small intestine, liver, lung, heart, kidney, gallbladder, xin bao or pericardium and san jiao) [3]. With the continuous understanding of TCM-HP, the TCM-HPs of bland and astringent were added into five fundamental tastes in Chinese pharmacopoeia (2015 edition), and it would further enrich and develop TCM-HP theory. Four toxic states were considered the toxic effects of TCM, and the toxic action will cause injury of organs and tissues, functional impairment, pathological changes and even death [2]. So in this research, we only considered the relationship between TCM actions and TCM-HPs (four fundamental character, five fundamental tastes, 12 meridians).

TCM-HPs are thus divided into three classes: character (C), taste (T), meridian (M) for this research and there are totally 24 TCM-HPs in the class of C, T, M, respectively. The 24 herbal properties can be further divided into 5, 7 and 12, which fall into C, T and M, respectively, as shown in Table 1. This study is based on the holism concept of TCM-HP to determine the classification for actions of TCM. Other 3 herbal properties (neutral, bland, astringent) were added based on classic concept of TCM-HPs. The strength of each TCM-HP is not considered, as this study is a qualitative one. The value of a specific TCM-HP is 1 if the herb possesses the corresponding property, and it is 0 if the herb does not possess the property [3].

**Table 1:** Chinese herbs properties’ binarization table of some HCHs and BASRHs

CHMs	V_1_	V_2_	V_3_	V_4_	V_5_	V_6_	V_7_	V_8_	V_9_	V_10_	V_11_	V_12_	V_13_	V_14_	V_15_	V_16_	V_17_	V_18_	V_19_	V_20_	V_21_	V_22_	V_23_	V_24_
Zu Ye	1	0	0	0	0	0	0	1	1	1	0	0	0	1	0	0	0	0	0	1	1	0	0	0
Qin Pi	1	0	0	0	0	0	1	0	0	0	1	0	1	0	0	0	0	0	1	0	0	1	0	0
Lian Qiao	1	0	0	0	0	0	1	0	0	0	0	0	0	1	0	1	0	0	0	1	0	0	0	0
Zhi Zi	1	0	0	0	0	0	1	0	0	0	0	0	0	1	0	1	0	0	0	0	0	0	0	1
Qing Hao	1	0	0	0	0	0	1	0	1	0	0	0	1	0	0	0	0	0	1	0	0	0	0	0
Huang Qin	1	0	0	0	0	0	1	0	0	0	0	0	0	0	1	1	0	0	1	1	0	1	0	0
Dan Shen	1	0	0	0	0	0	1	0	0	0	0	0	1	1	0	0	0	0	0	0	0	0	0	0

V_1_: cold,V_2_: cool, V_3_: neutral, V_4_: warm, V_5_: hot, V_6_: sour, V_7_: bitter, V_8_: sweet, V_9_: pungent, V_10_: bland, V_11_: astringent, V_12_: salty, V_13_: liver, V_14_: heart, V_15_: spleen, V_16_: lung, V_17_: kidney, V_18_: xin bao or pericardium, V_19_: gallbladder, V_20_: small intestine, V_21_: stomach, V_22_: large intestine, V23: bladder, V24: san jiao, respectively, each of which includes 5, 7 and 12 TCM-HPs. The total number of unique TCM-HP vector for all TCM is 5 + 7 + 12 = 24

For instance, the heat-clearing herb Huang Qin (*Scutellariae Radix*) has cold character, bitter taste; lung, gallbladder, spleen, large intestine and small intestine meridians entered. So the TCM-HP vector of Huang Qin (*Scutellariae Radix*) is **V** = [V_1_,V_2_,V_3_,V_4_,V_5_,V_6_,V_7_,V_8_,V_9_,V_10_,V_11_,V_12_,V_13_,V_14_,V_15_,V_16_,V_17_,V_18_,V_19_,V_20_,V_21_,V_22_,V_23_,V_24_] = [1,0,0,0,0,0,1,0,0,0,0,0,0,0,1,1,0,0,1,1,0,1,0,0] according to the order in Table 1.

### Traditional machine learning and deep learning methods

Machine learning explores the study and construction of algorithms that can learn from and make predictions on data [40]. The field of machine learning, which aims to build model from an example training set of input observations, and then make data-driven predictions or decisions expressed as outputs. The methods of machine learning hold promise to enable computers to assist humans in the analysis of large, complex data sets [41], and they are not following strictly static program instructions. Machine learning methods have been applied to a broad range of areas within genetics and genomics [7], drug discovery [42–44], medicinal and biomedical properties identification [45, 46], tracking literature [47], cancer risk prediction and diagnosis [48], wind power prediction [49], etc.

However, the success of machine learning systems often requires a large amount of labeled data which is expensive to obtain and significant manual feature engineering. These feature representations are often hand-designed, require significant amounts of domain knowledge and human labor, and do not generalize well to new domains [50].

Deep learning (DL), a concept closely associated with artificial neutral networks (ANNs), is in principle the learning of layered concepts. Thus, a model could describe higher and lower-level concepts at different layers of its structure [51]. Deep learning discovers intricate structure in large data sets by using the back propagation algorithm to indicate how a machine should change its internal parameters that are used to compute the representation in each layer from the representation in the previous layer [46]. Deep learning had been applied in adapting advanced neural network architectures for pharmaceutical research [14, 15, 52], predicting drug-induced liver injury (DILI) [17]. While deep learning and particularly unsupervised deep learning is still in its infancy, particularly in biological applications [53]. Moreover, deep learning network predicted drug property and activity with a relative accuracy improvement of approximately 14% over Merck’s in-house systems and resulted in an article in The New York Times [50, 54].

**Fig. 1:**
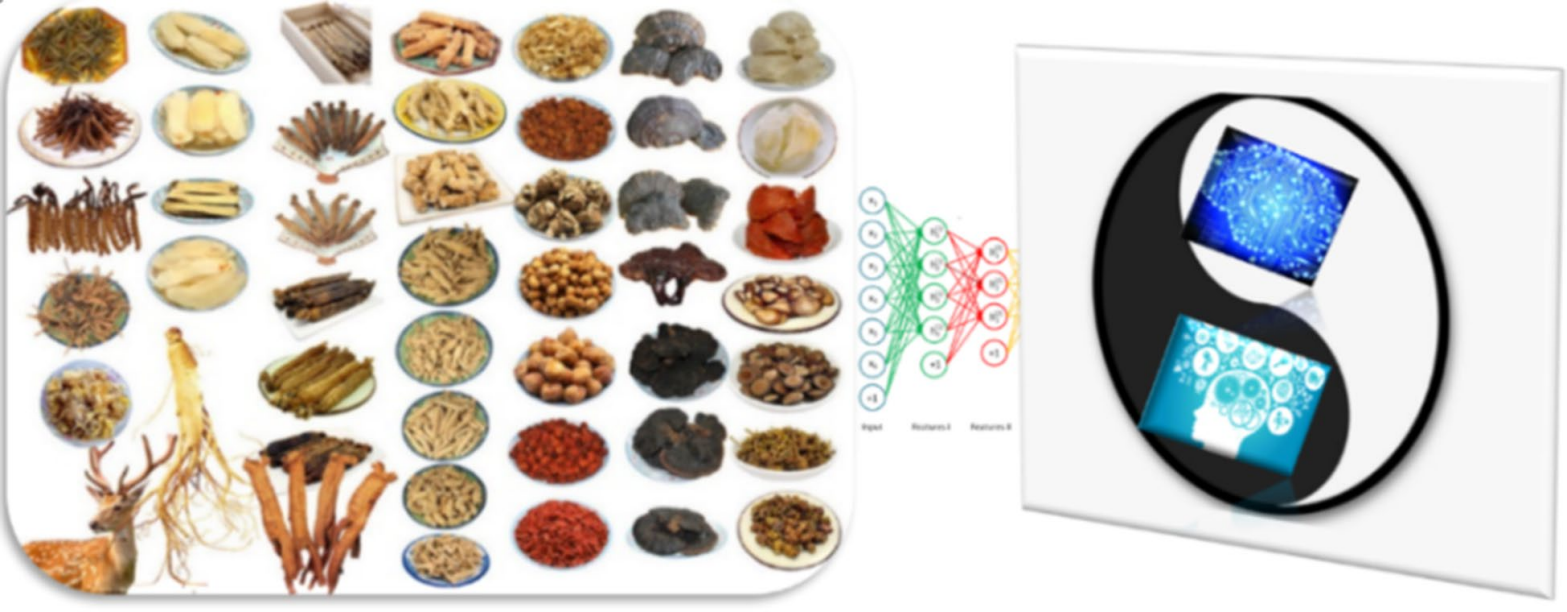
Interpretation of the scientific connotation behind the theory of TCM by deep learning methods. After TCM-HPs being converted to digital representation, they were entered as input vectors into the multi-layer neural networks. The output layer is the action classification with multiple processing layers to learn representations of TCM-HPs. We can excavate the underlying regularities and rules between TCM-HPs and actions with the deep neural networks architectures

The research of TCM-HP will be accelerated by using the deep learning methods, which will promote the intelligent study of TCM-HP. With the deep neural network architectures, we can excavate the underlying regularities and rules from the data recorded in ancient literature. As it is well-known that the data of traditional Chinese medicine is highly nonlinear, it is an inevitable trend to find out the inherent rules by using deep neural network (Fig. 1).

### Supported vector machine (SVM)

The support vector machine (SVM) is based on the statistical learning theory of VC dimension (Vapnik–Chervonenkis Dimension) and the risk structure of the minimum principle [55–58] and is also one of the most popular and successful binary classification methods. Its basic idea is to find a hyperplane in the feature space which separates the training data perfectly into two classes [59]. Moreover, SVM is a classier that performs classification tasks by constructing hyperplanes in a multidimensional space that separates cases of different class labels [60]. Least square support vector machine (LS-SVM) is an extension of standard support vector machines and it changes the error of optimization from first order into secondary order which covers a problem of solving quadratic programming into a problem of solving a set of linear equations [61, 62]. The method has been applied widely in biomedicine [63, 64].

### K-nearest neighbor (KNN)

K-nearest neighbor algorithm is the most widely used classification and clustering algorithm. The k-nearest neighbors (kNN) algorithm is one of the simplest machine-learning methods to understand and explain, and the principle being that an instance is classified by a majority vote of its neighbors [65]. It provides a simple and intuitive rule for pattern discrimination, which has resulted in its extensive use in a variety of applications and gains a high classification rate [66, 67].

**Fig. 2:**
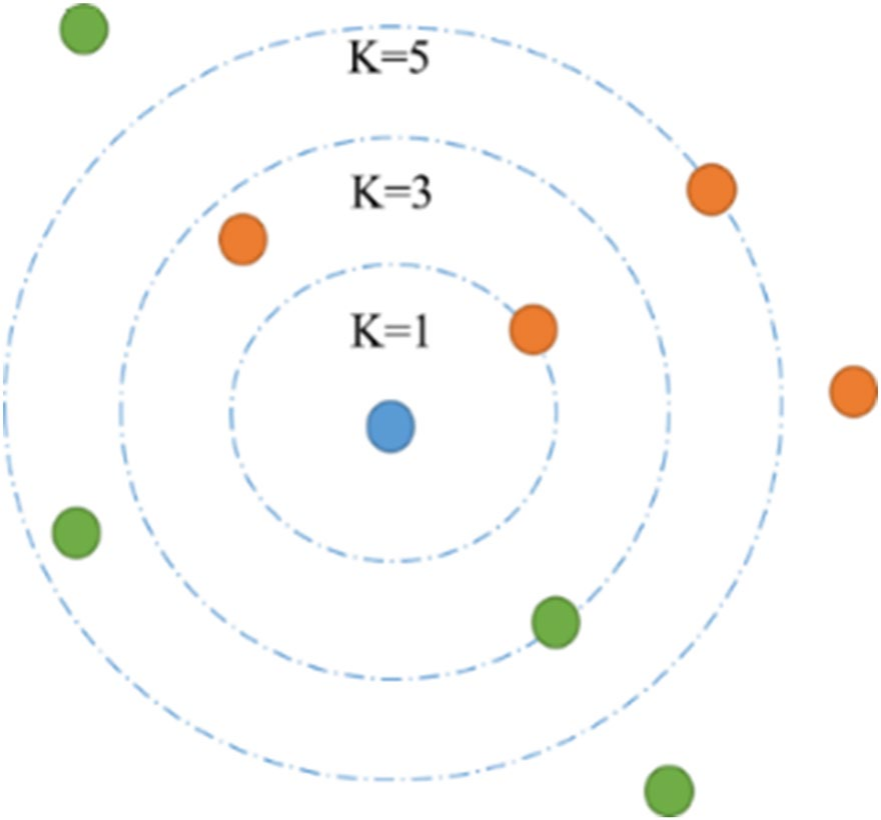
Illustration of a kNN classification model. For k = 3, the blue one will be assigned to the red class, this time by a 2-1 vote; however, the blue one will be classified into the green class by a 3–2 majority. The 24 TCM-HPs were considered as 24-dimensional vectors and Euclidean distance were used to compute any two Chinese herbal vectors distance. Chinese herbal actions classification are typically based on TCM-HPs and we can classify the two kinds herbs based on 24-dimensional vectors with the kNN

Each test sample is predicted to belong to the class most commonly found amongst its k closest neighbors, where k is a positive integer (Fig. 2). The Chinese herbal action classification is typically based on TCM-HP. TCM-HP is described as position vectors in the feature space that is usually of high dimensionality. Neighbors are identified on the basis of distance in the feature space. This is usually taken to be the Euclidean distance, though other metrics such as the Jaccard distance could be used. The minimum distance between the vectors gives the closest neighbor, so it is predicted that it belongs to the same class with the test object which the testing samples in the dataset are assigned to the class target value by a majority of its k nearest neighbor in the training set [68].

The Euclidean distance is often used to measure the similarity between two samples and more generally the distance between two p-dimensional vectors [69–71]. So the distance between these two TCM-HP vectors is computed as the length of the difference TCM-HP vector **V**_**r**_ − **V**_**s**_, denoted by

$$ d(V_{r} ,V_{s} ) = \left| {V_{r} - V_{s} } \right| = \sqrt {(V_{{r_{1} }} - V_{{s_{1} }} )^{2} + (V_{{r_{2} }} - V_{{s_{2} }} )^{2} \cdots + (V_{{r_{24} }} - V_{{s_{24} }} )^{2} } $$

where **V**_**r**_, **V**_**s**_ denotes the vectors of TCM **r** and **s**. The 24 TCM-HPs were considered as 24-dimensional vectors and Euclidean distance were used to compute any two Chinese herbal vectors distance.

### Deep belief network (DBN)

The deep belief network (DBN) is a neural network constructed from many layers of probabilistic model called restricted Boltzmann machines (RBMs) [72, 73].The training process of DBN can be achieved layer-by-layer from low to high layer to train these multilayer RBMs. Each RBM layer is trained by using the previous layer’s hidden units (h) as input/visible units (v). Moreover, one RBM has a single layer of hidden units which are not connected to each other and have undirected, symmetrical connections to a layer of visible units. Contrastive divergence [74] based pre-training of these RBM layers is carried out to initialize the weights of DBN. Then, using the gibbs sampling method, the unbiased sample set could be got.

Hinton et al. [73] used complementary priors and derived a fast, greedy algorithm that could learn deep, directed belief networks one layer at a time, provided the top two layers form an undirected associative memory. The fast, greedy algorithm was used to initialize a slower learning procedure that fine-tunes the weights using a contrastive version of the wake-sleep algorithm.

**Fig. 3:**
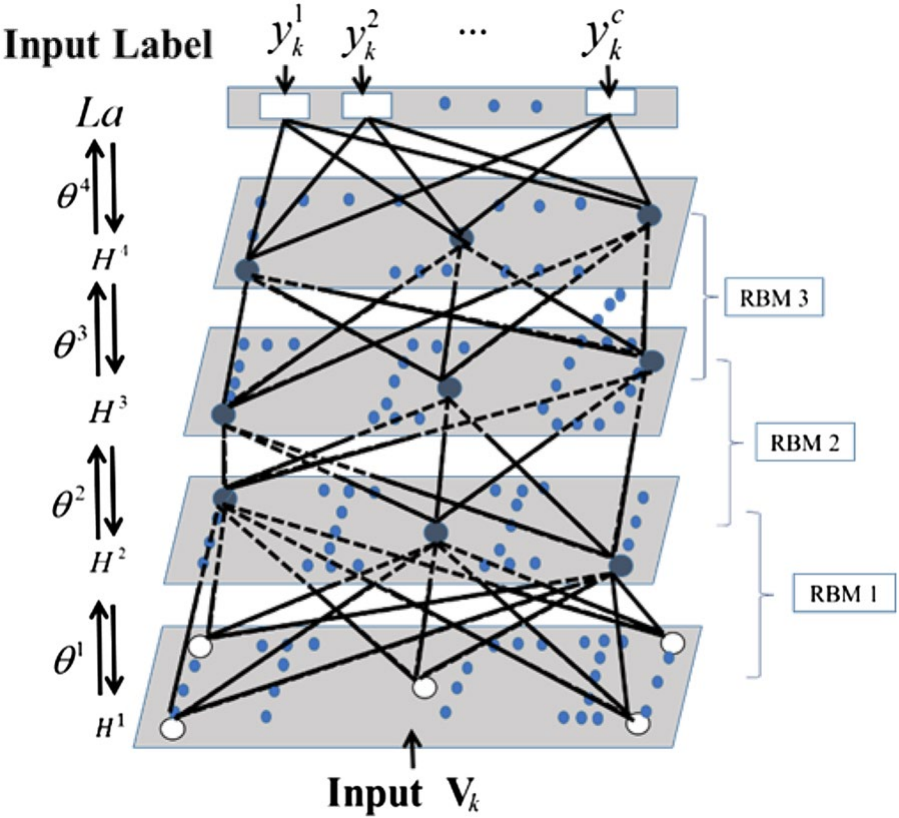
Schematic representation of a DBN. The number of layer and the number of units on each layer in the scheme are only examples. In this research, the TCM-HPs vectors were considered as input V_*k*_, and the action classification was considered as output label to train these multilayer RBMs

In order to solve the problem of scaling full-sized, high-dimensional in images recognition, multiresolution deep belief networks [75] and convolutional deep belief networks [76] were constructed. In this research, the TCM-HP vectors were considered as input V_*k*_, and the action classification was considered as output label as given in Fig. 3.

### Convolutional neutral network (CNN)

Convolutional networks combine three architectural ideas to ensure some degree of shift and distortion invariance local receptive fields, shared weights or weight replication and sometimes spatial or temporal subsampling. Convolutional process is the biologically inspired variant of multilayer perceptions (MLPs), which exploits the spatially local correlation by enforcing a local connectivity pattern [77, 78]. The classical convolutional network is composed of alternating layers of convolution and pooling (i.e. subsampling). The aim of the first convolutional layer is to extract patterns found within local regions of the input images that are common throughout the dataset [79].

**Fig. 4:**
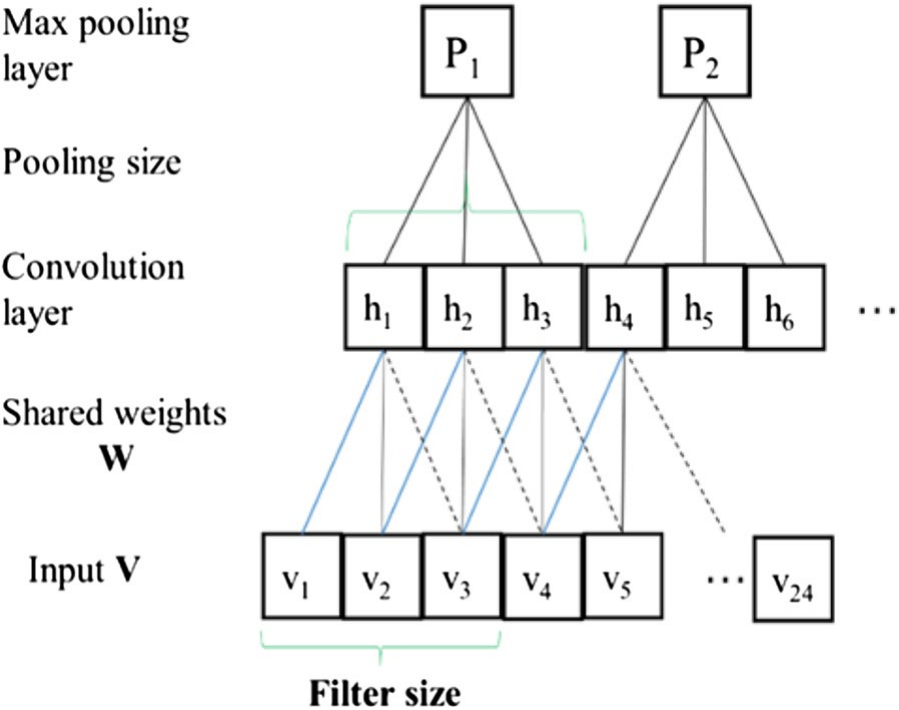
Diagram showing a typical convolutional network architecture consisting of a convolutional and max-pooling layer. In CNN, convolution layer is regarded as features extraction layer and each feature map is a mapping plane in feature map is a mapping plane in feature map layer. In our research, 24 TCM-HPs were entered as input vectors, convolution and pooling operations were then made for each TCM-HPs

In CNN, convolution layer is regarded as features extraction layer and each feature map is a mapping plane in feature map layer. The fully connected layers aggregate the local information learned in the convolutional layers to do class discrimination and fully-connected network like DNNs, each hidden activation h_*i*_ is computed by multiplying the entire input **V** by weights **W** in that layer [80]. The weights **W** are then shared across the entire input space, as indicated in Fig. 4. In our research, 24 TCM-HPs were entered as input vectors, convolution and pooling operations were then made for each TCM-HP.

### Methods for evaluating prediction performance of deep learning and traditional machine learning methods

For a binary classification exercise, predictions can be classed as true positives (TP), false positives (FP), true negatives (TN), and false negatives (FN). Cross-validation is also a popular strategy, and still allows models to be tested on data unseen in their generation.

As in the case of all discriminative methods, the performance of deep learning and traditional machine learning methods can be evaluated by the quantity of true positive or TP (correctly classified HCHs), true negative or TN (correctly classified BASRHs), false positive or FP (BASRHs falsely classified as HCHs), and false negative or FN (HCHs falsely classified as BASRHs) respectively. Sensitivity (P+), SEN = TP/(TP + FN) and specificity (P−), SPE = TN/(TN + FP) are the prediction accuracy for HCHs and BASRHs, respectively. The overall prediction accuracy, ACC = [(TP + TN)/(TP + TN + FP + FN)], and precision, PRE = TP/(TP + FP). The overall prediction accuracy and precision are used to measure the overall prediction performance.

The minimum standards of reporting checklist contains details of the experimental design, and statistics, and resources used in this study (Additional file 1).

## Results

### Distribution patterns of TCM-HPs of two kinds of herbs and their characteristics

**Fig. 5:**
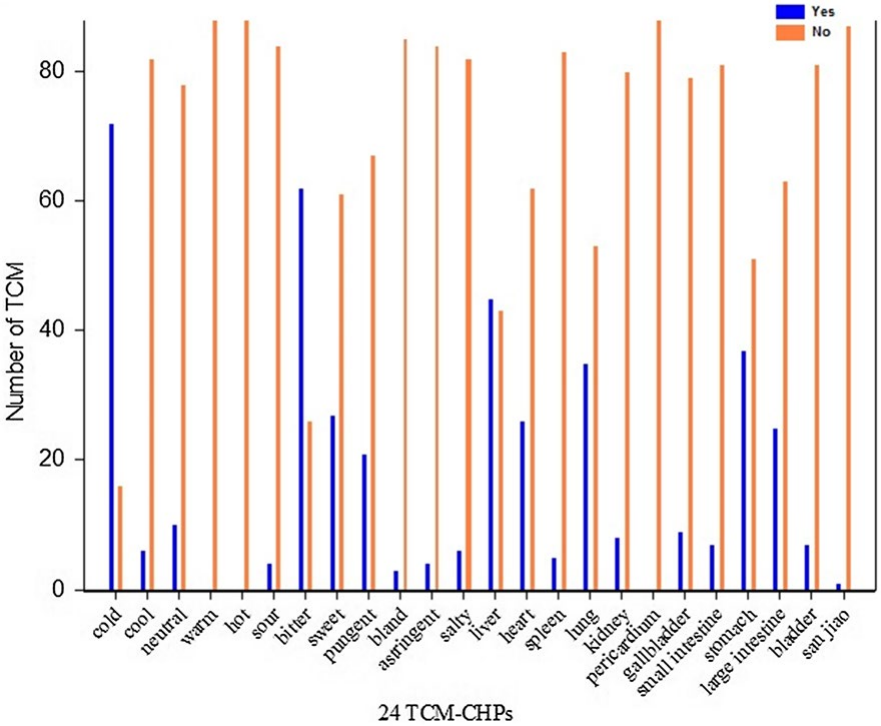
The TCM-HPs distribution of 88 HCHs. ‘Yes’ represents the herbs have the TCM-HP, and ‘No’ represents the herbs do not have this TCM-HP

**Fig. 6:**
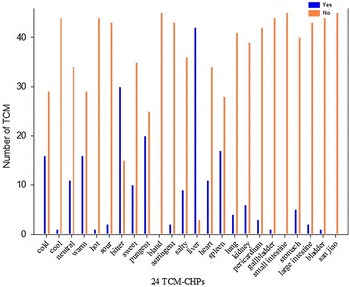
The TCM-HPs distribution of 45 BASRHs. ‘Yes’ represents the herbs have the TCM-HP, and ‘No’ represents the herbs do not have this TCM-HP

According to holistic view of the TCM-HPs, the properties of 88 known HCHs are predominantly cold characters, bitter taste; liver and stomach meridians entered, respectively, which are given in Fig. 5. The properties of 45 known BASRHs are predominantly warm characters, bitter and pungent taste; liver meridian entered, respectively, which are given in Fig. 6.

Figures 5, 6 showed the common distribution patterns of two kinds of herbs were bitter taste; liver meridian entered.

**Fig. 7:**
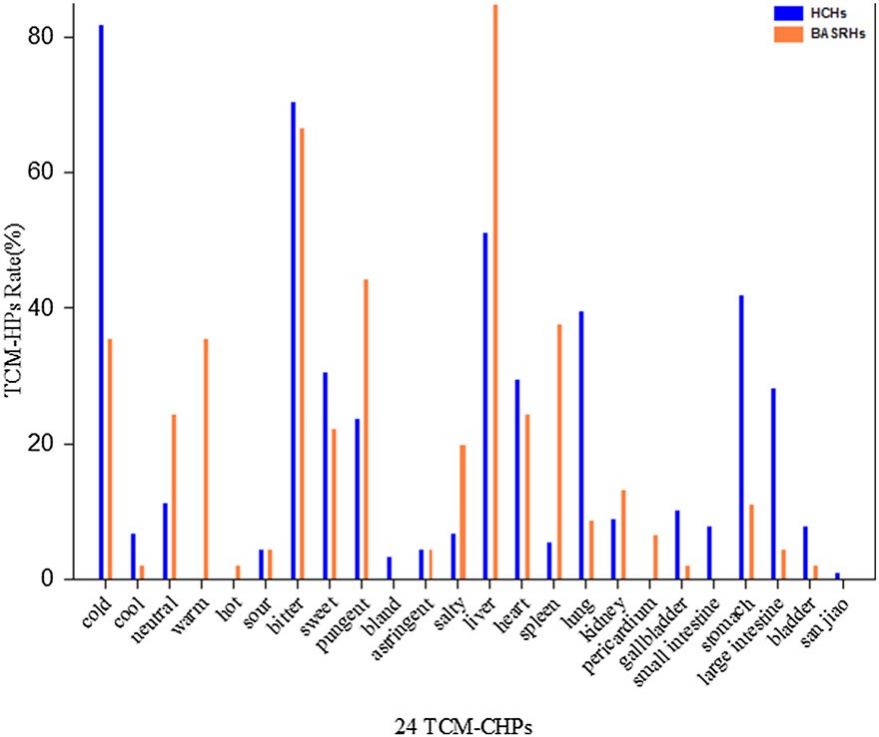
The TCM-HPs rate distribution of 88 HCHs and 45 BASRHs. TCM-HPs rate denotes that percentage of the HCHs (BASRHs) with the same TCM-HP in the total number of HCHs (BASRHs)

The TCM-HP rates of HCHs and BARSHs were compared as given in Fig. 7. From the herbal properties rate distribution, we knew that significant TCM-HP of BASRHs are bitter, pungent; liver entered and their rates were 66.7, 44.4, 93.3%, respectively. The prominent TCM-HP features were cold (81.8%), bitter (70.5%); liver (51.1%) and stomach (42.0%) entered in the 88 HCHs. Both of bitter and heart property rates in the two types of herbs were close proximity. However, the absolute value of differences for seven TCM-HP rates between HCHs and BASRH differed considerably as given in Table 2. If thirty percent of absolute value of difference was considered as setting value, the TCM-HP features were cold, warm character; spleen, liver and stomach meridians entered. Cold (81.8%)-bitter (70.5%)-liver (51.1%) combination could distinguish HCHs from BASRHs and warm (35.6%)-bitter (72.73%)/pungent (44.4%)-liver (93.3%) combination could distinguish BASRHs from HCHs.

**Table 2:** Seven TCM-HP rates of HCHs and BASRHs and their absolute values of difference between HCHs and BASRHs

TCM-HPs	HP rates of HCHs (%)	HP rates of BASRHs (%)	Absolute value of difference (%)
Cold	81.8	24.24	62.65
Warm	0.00	35.6	35.6
Pungent	23.9	44.4	20.5
Liver	51.1	93.3	42.2
Spleen	5.7	37.8	32.1
Stomach	42.0	11.1	30.9
Large intestine	28.4	4.4	24.0

### Models analysis of TCM-HPs for distinguishing HCHs from BASRHs

A cross-validation study was conducted to determine whether the traditional machine learning method SVM is able to separate HCHs and BASRHs based on their TCM-HPs. In this research, a SVM on the calibration set was constructed with setting coef 0 to 10, the highest number of polynomial kernel functions to 1. Polynomial was chosen as kernel function of this model and set tenfold cross validation when training this model. The sensitivity for the SVM model was 94.4%, and the specificity for this model was 72.4%, respectively. The overall prediction accuracy was 88.0%. The results on the validation set and external validation set were given in Table 3.

**Table 3:** Binary classification results with traditional machine learning and deep learning methods

Data set	Type of models	Sensitivity (%)	Specificity (%)	Precision (%)	Accuracy (%)
Calibration set	SVM	94.4	72.4	89.3	88
	DBN	/	/	/	/
	kNN	91.5	75.9	90.3	87
	CNN	/	/	/	/
Validation set	LS-SVM	82.4	81.3	82.4	81.8
	DBN	100	100	100	100
	kNN	82.4	62.5	70.0	72.7
	CNN	100	100	100	100
External validation set	SVM	93.3	75.0	87.5	85.0
	DBN	100	100	100	100
	kNN	86.7	80.0	92.9	85.0
	CNN	100	100	100	100

The best overall accuracy by kNN method was achieved 72.7% with k = 4 using the Euclidean distance on validation set and achieved 85.0% on external validation set.

A 100 hidden units RBM were constructed in this research, with setting batchsize to 5, learning rate to 2. After 10,000 iterations, the results showed that its sensitivity, specificity, precision, accuracy were 100.00, 100.0, 100.0, 100.0% on the validation set. On the external validation set, the same result were obtained with this DBN model.

In this research, we trained a 2 convolution layers and 2 subsampling layers convolutional neural network with setting batchsize to 5, learning rate to 0.001. In the first convolution layer, we set 3 output units and 2 kernels; and in the second convolution layer, we set 6 output units and 2 kernels. After 10,000 iterations, the model’s sensitivity, specificity, precision, accuracy were 100.00, 100.0, 100.0, 100.0% on the validation set and the external validation set.

The two deep learning method DBN, CNN achieved 100.0% accuracy on the validation set and external validation set than two other traditional machine learning methods was given in Table 3. It illustrated that deep learning methods had stronger predictive ability than two machine learning methods. These results also suggest that HCHs and BASRHs can be separated by deep learning and traditional machine learning methods based on their TCM-HPs with different accuracy and precision.

## Discussion

The herbal properties distribution of HCHs and BASRHs showed their differences on TCM-HPs. The TCM-HPs of 88 known HCHs are predominantly cold, bitter; liver and stomach meridians entered. The TCM-HPs of 45 known BASRHs are predominantly warm, bitter, pungent; liver meridian entered. The traditional machine learning methods and deep learning methods were adopted to construct the actions classification models based on the TCM-HP theory. The traditional machine learning methods SVM and kNN achieved 87.5, 92.9% overall prediction accuracy on external validation set. Furthermore, deep learning method DBN, CNN achieved 100.0% overall prediction accuracy. Two Chinese herbs (San Qi, Yin Xing Ye) were falsely classified as HCHs and Yu Gan Zi were falsely classified as BASRHs using SVM methods. In kNN model, two herbs (Niu Huang, Yin Xian Ye) were classified as HCHs and Yu Gan Zi (*Phyllanthus emblica* L.) was classified as BASRHs. Error classification CHMs with SVM and kNN on external validation set were given in Tables 4, 5.

**Table 4:** Error classification CHMs with SVM and kNN on external validation set

Category of CHMs	Type of models	Accuracy (%)	Error classification CHMs
HCHs and BASRHs	SVM	85.0	Yu Gan Zi (*Phyllanthus emblica* L.), San Qi (*Notoginseng radlx Et Rhizoma*), Yin Xian Ye (*Ginkgo folium*)
	kNN	85.0	Niu Huang (*Bovis calculus*), Yu Gan Zi (*Phyllanthus emblica* L.), Yin Xian Ye (*Ginkgo folium*)

**Table 5:** The herbal property of error classification CHMs

Error classification CHMs	TCM-HPs
*Yu Gan Zi* (*Phyllanthus emblica* L.)	Cool; sour, sweet, astringent; spleen, lung, stomach meridian entered
San Qi (*Notoginseng Radlx Et Rhizoma*)	Warm, bitter, sweet, liver and stomach meridian entered
Yin Xing Ye (*Ginkgo folium*)	Neutral, bitter, sweet, astringent; heart, lung meridian entered
Niu Huang (*Bovis calculus*)	Cool, sweet; heart, liver meridians entered

The 4 falsely classified CHMs using the traditional machine learning method SVM and kNN have the same characteristic in common—sweet. Furthermore, they are mostly liver and heart meridians entered. These results suggested that deep learning method DBN and CNN are capable of dividing known HCHs from known BASRHs and the TCM-HPs of the known HCHs contain useful information for distinguishing them from BASRHs.

## Conclusions

With deep learning methods and machine methods, we could understand the nonlinear relationship between TCM-HPs and actions. Moreover, the deep learning classification models would had better accuracy and generalization ability than machine learning in predicting actions of TCMs based TCM-HP theory. The distribution patterns of TCM-HPs between HCHs and BASRHs were analysed. HCHs were mainly cold, bitter; liver and stomach meridians entered. BASRHs are predominantly warm, bitter, pungent; liver meridian entered, respectively. Traditional machine learning and deep learning methods classification studies showed that HCHs could be distinguished from BASRHs based on TCM-HP theory.

Future work for elucidating the regularity of TCM formula compatibility using the deep learning methods based on TCM-HP theory was on going. This work will contribute to more specific and deeper understanding of the traditional Chinese medicine system. The expectation is that we can utilize the deeper regularity to guide the discovery of TCM, drug design and clinical treatment in the future.

## Additional files

**Additional file 1.** Minimum standards of reporting checklist.

**Additional file 2.** Raw data in Tables 1, 2, 3, 4 and 5.

**Additional file 3.** The raw data in Figs. 5, 6 and 7.

**Additional file 4.** The TCM-HP for HCHs and BARSHs.

**Additional file 5.** The codes of kNN, SVM, DBN, CNN.

## Abbreviations

TCM: traditional Chinese medicine
HPs: herbal properties
TCM-HPs: traditional Chinese medicine herbal properties
SVM: supported vector machine
kNN: k-nearest neighbor
DBN: deep belief network
CNN: convolutional neutral network
HCHs: heat-clearing herbs
BASRHs: blood-activating stasis-resolving herbs
DILI: drug-induced liver injury
DL: deep learning
ML: machine learning
